# Hydrocarbon divergence and reproductive isolation in *Timema* stick insects

**DOI:** 10.1186/1471-2148-13-151

**Published:** 2013-07-16

**Authors:** Tanja Schwander, Devin Arbuthnott, Regine Gries, Gerhard Gries, Patrik Nosil, Bernard J Crespi

**Affiliations:** 1 Center for Ecological and Evolutionary Studies, University of Groningen, Groningen, Netherlands; 2 Department of Ecology and Evolution, University of Lausanne, Lausanne, Switzerland; 3 Department of Biology, University of Ottawa, Ottawa, Canada; 4 Department of Biological Sciences, Simon Fraser University, Burnaby, Canada; 5 Department of Animal and Plant Sciences, University of Sheffield, Sheffield, UK

## Abstract

**Background:**

Individuals commonly prefer certain trait values over others when choosing their mates. If such preferences diverge between populations, they can generate behavioral reproductive isolation and thereby contribute to speciation. Reproductive isolation in insects often involves chemical communication, and cuticular hydrocarbons, in particular, serve as mate recognition signals in many species. We combined data on female cuticular hydrocarbons, interspecific mating propensity, and phylogenetics to evaluate the role of cuticular hydrocarbons in diversification of *Timema* walking-sticks.

**Results:**

Hydrocarbon profiles differed substantially among the nine analyzed species, as well as between partially reproductively-isolated *T*. *cristinae* populations adapted to different host plants. In no-choice trials, mating was more likely between species with similar than divergent hydrocarbon profiles, even after correcting for genetic divergences. The macroevolution of hydrocarbon profiles, along a *Timema* species phylogeny, fits best with a punctuated model of phenotypic change concentrated around speciation events, consistent with change driven by selection during the evolution of reproductive isolation.

**Conclusion:**

Altogether, our data indicate that cuticular hydrocarbon profiles vary among *Timema* species and populations, and that most evolutionary change in hydrocarbon profiles occurs in association with speciation events. Similarities in hydrocarbon profiles between species are correlated with interspecific mating propensities, suggesting a role for cuticular hydrocarbon profiles in mate choice and speciation in the genus *Timema*.

## Background

Individuals in natural populations commonly prefer certain trait values over others when choosing their mates [1,2]. Traits that are used for mate selection include vocal signals (e.g., [3,4]), pheromones (e.g., [5]), behavioral repertoires (e.g., [6]), and morphological compatibility (e.g., [7,8]). Theory predicts that divergent preferences for certain trait values can generate behavioral reproductive isolation at both the intra- and interspecific levels [9-11]. Consistent with this prediction, mating preferences have been observed to vary among populations and closely related species in nature (e.g., [1,12,13]), and in multiple taxa evidence suggests that the resulting behavioral isolation has been involved in speciation [11].

Species-specific mating preferences can arise via different mechanisms, and one of the critical components to understanding the process of speciation is determining which factors promote divergence in traits used for mate selection. For example, if mating with individuals from other populations is associated with costs, selection for population-level recognition can drive the evolution of mating signals (e.g., [1,8,14]). The resulting behavioral isolation can promote speciation and may lead directly to increased levels of prezygotic isolation, as via reinforcement [11,15]. Alternatively, differences in mate selection traits between species can arise via neutral processes [16,17], as a by-product of divergent ecological adaptation [13,18], or they can evolve in parallel with reproductive isolation, for example as a consequence of sexual selection. Under these scenarios, the same forces of evolution that affect signal properties for intra-specific mate choice would also affect reproductive isolation between species.

The different mechanisms that can cause species-specific mating preferences are expected to generate distinct patterns of evolutionary trait divergence [19]. If mating preferences contribute to the evolution and maintenance of reproductive isolation among species, mating signals are expected to change rapidly during the speciation process. Little change might occur between speciation events, because stabilizing selection for reliable species discrimination should decrease variance in mating signals [20,21]. Stabilizing selection for species discrimination should also favor large signal differences between species relative to intraspecific variation [22,23]. By contrast, under sexual selection or drift, mating signals may be expected to change continuously over evolutionary time, without accelerated change during periods of species formation [1,24]. Thus, distinguishing between gradual vs. speciation-associated change of mating signals in a taxon can provide insights on the selective forces underlying phenotypic change [8,19,25].

Here, we investigate whether cuticular hydrocarbons may provide a signal for mate recognition in *Timema* stick insects, and whether hydrocarbon profiles have diverged gradually between species or if most change is associated with speciation events. Chemical communication among insects is extremely widespread (e.g., [26-28]), and cuticular hydrocarbons, in particular, serve as mate recognition signals in many species (reviewed in [29]). Analyses of courtship behavior suggested that chemical signals also underlie species recognition and premating isolation in *Timema* stick insects [30], a genus that comprises 21 described species (16 of them sexual, the others asexual) of plant-feeding insects, distributed primarily in California [31]. In *Timema*, sexual isolation has been shown to represent an important reproductive barrier separating ecologically isolated populations within, as well as between, species [30,32,33].

We analyzed cuticular hydrocarbon components of nine closely-related sexual species of *Timema* (*T*. *bartmani*, *T*. *boharti*, *T*. *californicum*, *T*. *chumash*, *T*. *cristinae*, *T*. *knulli*, *T*. *petita*, *T*. *podura*, and *T*. *poppensis*) to characterize differences among species and to test whether interspecific matingsare more likely between species with similar than distinct hydrocarbon profiles. Using the phylogeny of these species, we also evaluated the tempo and mode of hydrocarbon profile evolution to develop insights into the processes underlying divergence between species for this trait. Finally, in order to test for possible links between species recognition, intraspecific mate choice, and the early stages of the speciation process in *Timema*, we tested for divergence in hydrocarbon components between populations within one of the nine species, *T*. *cristinae*. This species was chosen because there is extensive evidence for sexual isolation between populations occurring on *Ceanothus* versus *Adenostoma* host plants (e.g., [33-35]) but the proximate mechanisms underlying mate discrimination have remained speculative. This analysis allows us to assess whether the same traits that are used to distinguish conspecific from heterospecific individuals may also be used in mate choice within species.

## Results

### Cuticular hydrocarbon variation between species and populations

For each of the nine species, we quantified the amount of five cuticular hydrocarbon components (3-, 5-, 7-, 9/11- and 13-methylheptacosane, henceforth 3Me27, 5Me27, 7Me27, 9/11Me27, and 13Me27, respectively) of three to 11 virgin females. Because our approach required analyzing nine species in parallel, we focused on only one sex for simplicity, and we used females because a previous study indicated that mate discrimination, at least in its early stages, appears to mainly depend on males. Specifically, males are more likely to pair with, and court, females of their own than of other species [30]. For eight of the nine species we used females from two geographically distant locations, to account for intraspecific variation when evaluating species differences (Table 1). Except for *T*. *bartmani* and *T*. *petita*, which are known to occur only on a single host plant (*Abies concolor* and *Ceanothus spp*, respectively), the two populations of each species were collected on different hosts (Table 1). For the ninth species, *T*. *boharti*, we were able to collect individuals from only one location; across the nine species, we therefore included individuals from 17 locations in total. Sixteen of these 17 locations comprised only the focal species, with no other *Timema* species present on the same or different host plants. For one of the populations (ED, Table 1), the focal species *T*. *chumash* overlapped with a second species, *T*. *podura*. Because past work implicates reinforcement of mating preferences between co-occurring populations of *T*. *cristinae *[35], we specifically chose isolated populations to avoid potentially increased levels of discrimination at locations where species meet.

**Table 1:** **Sampling locations and host plant information for interspecific analyzes**, **as well as for intraspecific comparisons in *T***. ***cristinae***

**Species**	**Population**	**Hostplant**	**Coordinates**	
*T*. *bartmani*	JL	*Abies concolor*	34.170000	-117.002017
*T*. *bartmani*	RS	*Abies concolor*	34.210000	-117.098333
*T*. *boharti*	ML	*Adenostoma*	32.881200	-116.445917
*T*. *californicum*	Fremont	*Adenostoma*	36.763517	-121.502533
*T*. *californicum*	HW1	*Quercus*	36.286933	-121.841883
*T*. *chumash*	HW2	*Adenostoma*	34.269700	-118.168483
*T*. *chumash*	ED	*Ceanothus*	33.885183	-116.859783
*T*. *cristinae*	WTA	*Adenostoma*	34.515833	-120.073150
*T*. *cristinae*	OJ	*Ceanothus*	34.485300	-119.298117
*T*. *knulli*	Trail	*Sequoia*	35.836200	-121.391483
*T*. *knulli*	BC	*Ceanothus*	36.071017	-121.595567
*T*. *petita*	Ma	*Ceanothus*	36.358900	-121.900350
*T*. *petita*	Mo	*Ceanothus*	36.476533	-121.936133
*T*. *podura*	HW243	*Adenostoma*	33.815117	-116.791033
*T*. *podura*	Seq	*Ceanothus*	35.583333	-118.533333
*T*. *poppensis*	Fish	*Pseudotsuga*	38.885283	-123.517150
*T*. *poppensis*	Mado	*Sequoia*	36.996433	-121.717783
*T*. *cristinae*	OGA	*Adenostoma*	33.990567	-116.061417
*T*. *cristinae*	R23A	*Adenostoma*	34.519067	-120.077500
*T*. *cristinae*	SC	*Ceanothus*	34.522300	-119.831283
*T*. *cristinae*	PEC	*Ceanothus*	34.491333	-119.795017

**Figure 1:**
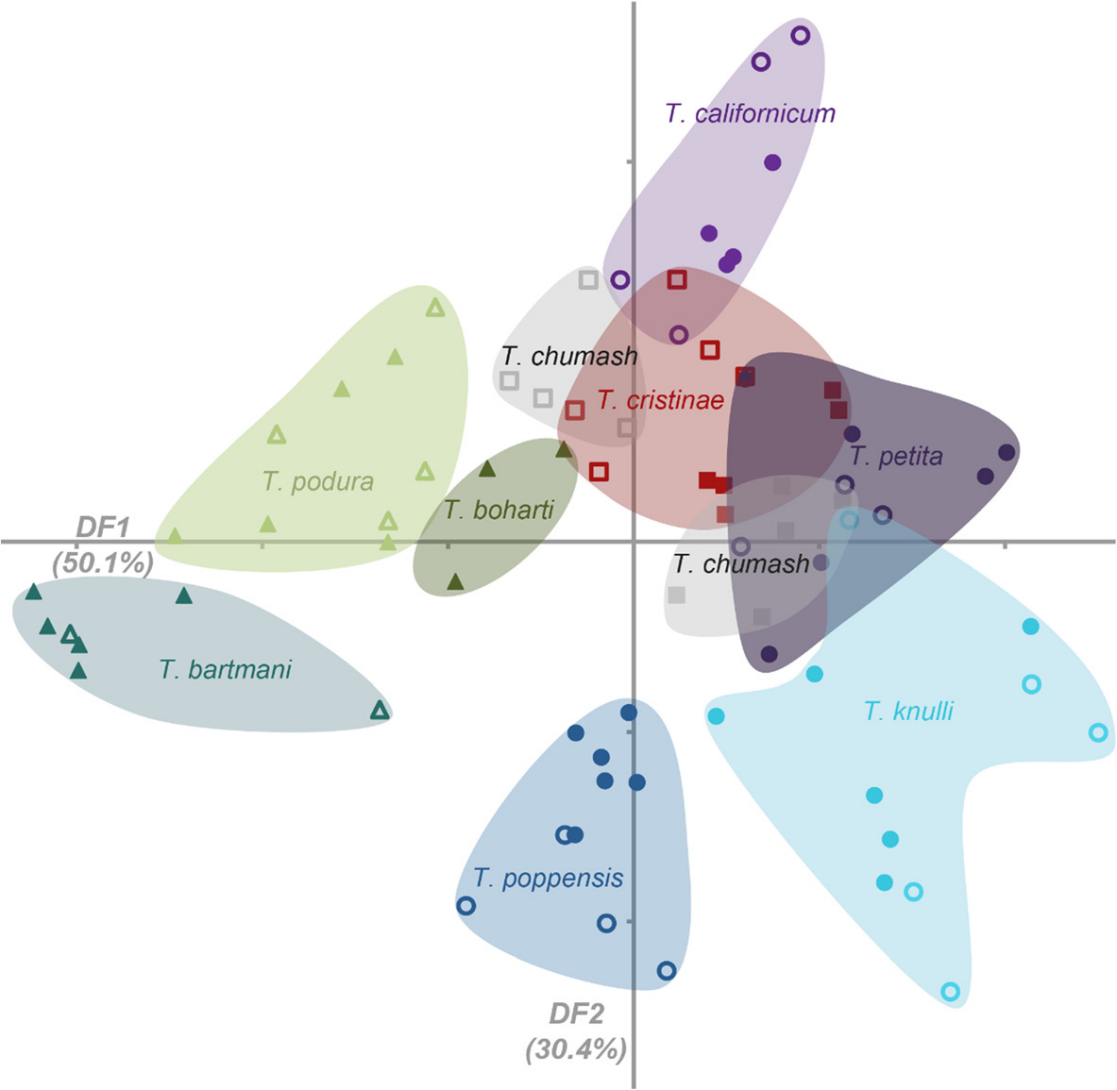
***Timema *species are characterized by distinct cuticular hydrocarbon profiles.** Five different hydrocarbon components determined for 3–11 individuals per species are log-contrast transformed and summarized via the first two discriminant functions (DF1, DF2), explaining respectively 50.1% and 30.4% of hydrocarbon variation between species. Individuals from different populations within each species are distinguished by open vs closed symbols (between population divergence is significant for *T*. *chumash*, *T*. *cristinae* and *T*. *poppensis*).

**Figure 2:**
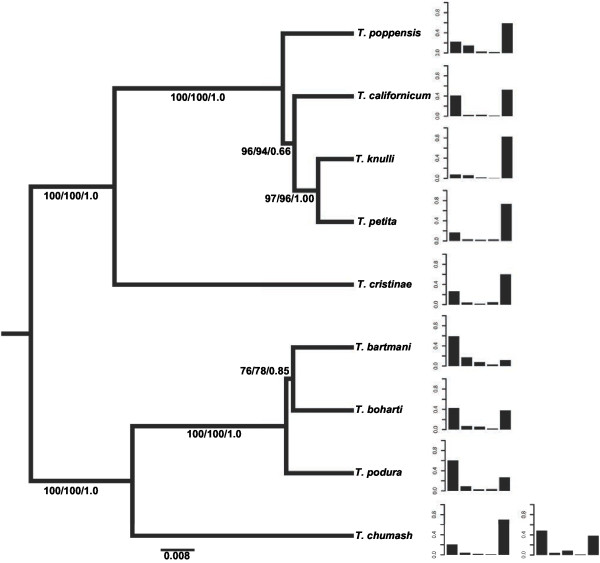
**Inferred species phylogeny** (**based on 1879 bp concatenated nuclear and mitochondrial sequences**) **and hydrocarbon profiles for each species.** The height of the bars in each profile indicates the average relative amounts of the different hydrocarbon sets (left to right): 13Me27, 9Me27 + 11Me27, 7Me27, 5Me27 and 3Me27 (see footnote in Table 3). The components 3Me27 and 13Me27 are characterized by strong phylogenetic autocorrelation (see text for details). Because in *T*. *chumash*, individuals from the two analyzed populations profiles are characterized by highly divergent hydrocarbon profiles, separate profiles are depicted for each population (left: population HW2, right: ED). Populations are pooled for the other species’ profiles. Numbers associated with branches in the phylogeny indicate branch support values (bootstraps) from the ML and parsimony analyzes, as well as Bayesian posterior probabilities, respectively.

In a first step, we tested whether *Timema* species differed in the composition of their cuticular hydrocarbons, as expected if these chemicals contribute to sexual isolation. Accordingly, a non-parametric MANOVA revealed significant hydrocarbon profile differences among *Timema* species (populations nested within species; species effect: *F*_*8*,*8*_ = 10.4, *p* <0.0001, populations within species: *F*_*8*,*59*_ = 3.8, *p* <0.0001; Figure 1). To simplify the graphical representation of hydrocarbon profile differences between species, we combined the hydrocarbon components using discriminant function analysis, after log-contrast transformation [14,36]. Log contrasts were calculated by dividing the value for each hydrocarbon by the component 13Me27, and then taking the log of these new variables, resulting in four log-contrast transformed values [log-contrasts for 3Me27, 5Me27, 7Me27 and 9/11Me27, referred to as LC3 (Log Contrast 3), LC5, LC7 and LC9-11, respectively] for every individual. Results using other components as the divisor were qualitatively similar. The two first discriminant functions (DF1, DF2) captured 50.1% and 30.4% of the total variance between species, respectively (loadings: DF1= −2.95 × LC3 + 0.03 × LC5 + 0.01 × LC7 + 5.8 × LC9-11; DF2= −5.60 × LC3 - 0.34 × LC5 + 0.63 × LC7 + 0.10 × LC9-11). DF1 and DF2 also encompass sufficient variance to allow for the visual separation of species (Figure 1). While the between-population differences in hydrocarbon profiles were relatively small compared to the between-species differences (78.0% of the variation between species vs. 7.5% between populations within species), *T*. *chumash* stood out with individuals from the two populations being characterized by very distinct hydrocarbon profiles (Figure 1, MANOVA: Wilks’ *λ* = 0.02, *F*_*1*,*7*_ = 65.4, *p* = 0.0007). This difference in *T*. *chumash* is consistent with previous data on notable between-population divergence in chromosome numbers [37], and mtDNA (Crespi, unpubl. data). It is unlikely to represent a profile shift in areas of overlap with other species as the hydrocarbon profiles of *T*. *chumash* individuals in areas of overlap with *T*. *podura* (population ED) appeared more similar to *T*. *podura* than the profile of individuals from the isolated *T*. *chumash* population (population HW2; Figure 2). Significant hydrocarbon profile differences of much smaller effect size were also found between the two populations of the species *T*. *cristinae* (Wilks’ *λ* = 0.09, *F*_*1*,*8*_ = 13.0, *p* = 0.007) and *T*. *poppensis* (Wilks’ *λ* = 0.18, *F*_*1*,*8*_ = 5.6, *p* = 0.04) but not for any of the remaining species (*T*. *bartmani*, *T*. *californicum*, *T*. *knulli*, *T*. *petita*, or *T*. *podura*, all p > 0.27).

### Hydrocarbon variation and between-species mating propensities

Next, we tested whether hydrocarbon profile differences may be involved in interspecific mating decisions by pairing individual males and females and recording whether mating occurred. These no-choice mating trials (> 1400 in total, Table 2) revealed large-scale differences among species combinations in their propensity for interspecific matings, ranging from 0% to almost 95%. Some of this variation appeared to stem from variation among species in male “eagerness” for mating. Males of some species courted any female shortly after encountering her, whereas males from other species were reluctant to court females of even their own species. This pattern is reflected by the significant differences among species in mating proportions measured for intraspecific no-choice trials (*χ*^2^= 55.3, df = 8, p < 0.0001, range 0.42 to 1.00) as well as by the fact that the interspecific mating proportions measured for the reciprocal species combinations were only moderately correlated (Pearson's product–moment correlation: rho= 0.45; t = 2.9, df = 33, p = 0.007). We therefore analyzed the interspecific mating propensities in two ways (1) using the average values of the reciprocal combinations for each species pair, and (2) using two separate points for each species pair and using male species as the first explanatory factor in the statistical analyses (based on behavioral evidence for a strong male effect on pairing and mating [30]).

**Table 2:** Number of mating trials per species combination

	**Male**								
**Female**	***T. bartmani***	***T. boharti***	***T. californicum***	***T. chumash***	***T. cristinae***	***T. knulli***	***T. petita***	***T. podura***	***T. poppensis***
*T*. *bartmani*	30	4	9	12	25	3	3	13	25
*T*. *boharti*	8	4	35	13	13	4	8	20	13
*T*. *californicum*	11	4	22	11	27	13	9	30	41
*T*. *chumash*	10	3	16	19	19	19	20	34	26
*T*. *cristinae*	10	4	28	15	106	12	10	26	30
*T*. *knulli*	9	3	7	12	20	17	16	22	18
*T*. *petita*	11	2	19	6	28	21	8	20	21
*T*. *podura*	24	12	3	24	31	4	18	20	15
*T*. *poppensis*	10	11	28	10	24	24	8	26	53

Both types of approaches are consistent with the idea that cuticular hydrocarbon profiles function as signals for interspecific mate discrimination in *Timema*. The mean interspecific mating proportions were significantly negatively correlated with hydrocarbon profile differences between species (as measured by species distances in the multivariate hydrocarbon component space; Mantel’s r: -0.43, p= 0.001). Interspecific mating propensities were also negatively correlated with mitochondrial genetic divergences between species (Mantel’s r: -0.53, p= 0.002). The correlation between hydrocarbon profile differences and mating propensity was not, however, explained by genetic divergences, as these two variables were not significantly correlated with each other (Mantel’s r: 0.22, p= 0.09), and hydrocarbon profile differences were still negatively correlated with interspecific mating propensities after correcting for genetic divergences between species (partial Mantel: r = −0.39, p= 0.002).

We obtained qualitatively the same results when using each male–female species combination as a separate data point and using male species as an explanatory variable (to correct for male “eagerness”). Thus, interspecific mating propensity was significantly affected by cuticular hydrocarbon profile divergence, even after correction for genetic divergence (glm with quasibinomial error distribution; effect of genetic divergence: t= −5.5, p< 0.0001, hydrocarbon divergence: t= −2.9, p= 0.006). However, the significance values from these analyses must be considered with caution due to the non-independence of the different pairwise species divergences.

To develop insights into possible contributions of individual hydrocarbon components to the association between interspecific mating propensity and hydrocarbon profile divergences, we also tested for correlations between mating proportions and differences between species in their levels of each individual hydrocarbon (i.e., the five non log-transformed hydrocarbon components). Differences for 3Me27, 7Me27, and 13Me27 were significantly negatively correlated with interspecific mating propensity (Mantel’s r: -0.30 to −0.51, all p<0.02), whereas the two other components were not or only marginally non-significantly correlated with interspecific mating propensity (5Me27:r= 0.13, p = 0.77; 9Me27 + 11Me27: r= −0.24, p = 0.06). These patterns indicate that putative mating cues would likely be derived from global or combinatorial profiles rather than individual components, and/or that different components are used as signals by different species.

### Macroevolutionary change in CHCs

Finally, we wanted to infer whether cuticular hydrocarbon profile differences between species develop gradually over time, as expected under constant intraspecific selection or neutral divergence, or whether there is accelerated change during speciation events. To this end, we first built a maximum-likelihood (ML) phylogeny describing the relationships between the nine *Timema* species used for hydrocarbon analysis (Figure 2). The branch lengths in this phylogeny can be interpreted as proportional to time, as the ML tree with a global clock constraint did not differ significantly from the best (unconstrained) ML tree (likelihood ratio test: p = 0.15). Maximum parsimony analyses and Bayesian inferences yielded the same tree topology (Figure 2), which is fully compatible with previous phylogenies of the genus [38,39].

We then used this phylogeny to investigate the evolutionary tempo and mode of hydrocarbon profile divergence, represented by DF1 and DF2, as well as of the individual hydrocarbon components. In particular, we inferred whether hydrocarbon profiles tend to diverge continuously between species, or whether the amount of profile changeis correlated with the numberof speciation events [19]. To this end, we evaluated the fit of nine different diffusion-based maximum-likelihood models, each representing a specific evolutionary scenario of character change. These scenarios include a 'neutral divergence' model whereby character change occurs on all branches in the phylogeny and is proportional to time, as well as different types of 'speciation' models where character change coincides with species splits (nodes) in the phylogeny (see Table 3 for a summary of models and interpretations and Methods and Additional file 1 for details). Akaike Information Criterion (AIC) values are calculated to determine which of the nine models of evolutionary change best characterizes the tempo and mode of diversification of hydrocarbon profiles in *Timema*.

**Table 3:** **Summary of the nine character change models**, **and the fit of each model to *Timema *hydrocarbon profiles** (**represented by DF1 and DF2**) **and individual hydrocarbon components**

**Model for trait change**	**P**^**1**^	**Interpretation**	**Conclusion if best fit**	**Profiles**		**Hydrocarbon components**				
				**DF1**	**DF2**	**3Me27**	**5Me27**	**7Me27**	**9Me27 + 11Me27**	**13Me27**
Pure-Phylogenetic/Distance	1	Time predicts the amount of change occurred (consistent with neutral divergence).	Trait not involved in speciation	2.1	5.6	*	2.1	2.3	2.9	*
Pure-Phylogenetic/Equal	1	The amount of change depends on the number of speciation events occurred (number of nodes).	Consistent with speciational change^2^	2.0	3.4	**0.5**	2.4	*	**1.7**	**0.1**
Pure-Phylogenetic/Free	16	Trait values can change at any rate between speciation events	Trait not involved in speciation	24.7	27.0	34.9	44.2	40.9	29.0	27.6
Nonphylogenetic/Distance	1	Closely related species share trait values for a short time and then diverge very rapidly	Trait not involved in speciation	8.0	2.0	7.0	*	5.5	**1.5**	5.4
Nonphylogenetic/Equal	1	Trait values change very rapidly, with similar rates in different lineages	No inference^3^	5.4	*	4.3	**0.1**	3.9	*	2.6
Nonphylogenetic/Free	9	Trait values change very rapidly, with different rates in different lineages	No inference^3^	12.8	14.8	2E+08	2E+08	2E+08	2E+08	2E+08
Punctuated/Distance	1	At each speciation event, one daughter species retains the ancestral trait value, the trait in the other daughter species changes, with the amount of change dependent on time	Consistent with speciational change^2^, level of divergence between species also affected by the time separating them	**1.1**	5.0	5.3	14.2	17.3	12.7	6.4
Punctuated/Equal	1	At each speciation event, one daughter species retains the ancestral trait value, the trait in the other daughter species changes, the amount of change between speciation events is always the same	Consistent with speciational change^2^	*	**0.9**	5.4	13.9	12.3	9.0	5.3
Punctuated/Free	8	At each speciation event, one daughter species retains the ancestral trait value, the trait in the other daughter species changes, the amount of change between speciation events varies freely	Consistent with speciational change^2^, level of divergence between species also affected by lineage-specific processes	15.1	19.0	19.3	28.2	31.3	26.7	20.4

Model-fit values are presented as AIC value differences between the focal model and best model (labeled with *). Models that differ by at least 2 AIC units from the best model are considered to provide a significantly worse fit to the data; models within the 2 units are indicated in bold. 3Me27 = 3-methylheptacosane; 5Me27 = 5-methylheptacosane; 7Me27 = 7-methylheptacosane; 9Me27 = 9-methylheptacosane; 11Me27 = 11-methylheptacosane; 13Me27 = 13-methylheptacosane. Notice that the convergent results for 3Me27 and 13Me27 are expected given the strong negative correlation between these two components. 1: Numbers of parameters estimated; see Additional file 1 for details 2: While these models are expected to be the best description for traits involved in speciation, similar phylogenetic patterns may also arise via other processes, for example, when speciation and trait evolution are associated with niche shifts [18]. 3: Support for these models indicates very rapid divergence of traits; however, they do not provide insights into why such rapid divergence occurs (e.g., genetic drift, natural selection).

The evolution of global hydrocarbon profiles (described by DF1 and DF2) were better explained by change occurring proportionally to speciation events (nodes in the phylogeny) than by neutral models in which change occurs proportionally to time (branch lengths). Specifically, the evolution of DF1 on the *Timema* phylogeny was best explained by punctuated models, in which at each node, one daughter branch retains the ancestral character value, and the other daughter branch changes(AIC differences between punctuated and other models>2; Table 3). For DF2, models with equal branch lengths provided a better fit to the data than any other model, but it was not possible to distinguish between nonphylogenetic and punctuated models (AIC differences <2; Table 3). The evolutionary patterns returned by the analyses for individual components varied widely. Support for speciational change was found for one of the five analyzed hydrocarbon components, 7Me27, where a phylogenetic model with equal branch lengths provided the best fit to the data (Table 3). Phenotypic change was therefore dependent on the number of speciation events but independent of time since divergence. By contrast, we found that evolutionary change in 3Me27 was best described by a pure-phylogenetic model, with change proportional to branch lengths, consistent with the significant correlation of LC3 differences between species and their genetic divergences. The same pattern was also revealed for 13Me27, as expected given the strong correlation between 13Me27 and 3Me27 (Pearson's product–moment correlation: rho= −0.95; t = −26.2, df = 77, p < 0.0001). The evolutionary change of the two remaining components (5Me27 and 9Me27 + 11Me27) was best described by non-phylogenetic models, which provide little information on processes driving character changes (Table 3). These diverse patterns for individual components could indicate that different species use different components as cues, or, more likely, that combinations of several components are used for species discrimination. The latter hypothesis is notably supported by DF1, representing a profile combination, and being best described by punctuational (i.e., speciational) models of change.

Altogether, our data thus indicate that cuticular hydrocarbon profiles vary among *Timema* species, and that most evolutionary change in global hydrocarbon profiles occurs in association with speciation events. Individual hydrocarbon components appear to change according to different scenarios, with some following patterns expected under neutral evolution and others with the amount of change more strongly correlated with speciation events than with divergence time. Similarities in hydrocarbon profiles between species are correlated with inter-specific mating propensities which in combination with the punctuational change of DF1 and DF2 on the *Timema* phylogeny supports the idea that hydrocarbon profiles may function as inter-specific mating signals in this group.

### *Cuticular hydrocarbon variation among* T. cristinae *populations*

To test whether cuticular hydrocarbon profiles are also correlated with mating decisions at the intraspecific level, we combined an additional set of four populations of the species *T*. *cristinae* with the two *T*. *cristinae* populations included in the interspecific analyzes. This species was chosen because there is extensive evidence for host-plant associated mating preferences, and partial reproductive isolation, between populations occurring on *Ceanothus* host plants and populations occurring on *Adenostoma* host plants (e.g., [33-35]), but the proximate mechanisms underlying such mate discrimination have remained speculative.

A MANOVA revealed significant cuticular hydrocarbon profile differences between populations on different host plants but also differences between populations within hosts (populations nested in hosts; host effect: Wilks’ *λ* = 0.63, approx. *F*_*1*,*4*_ = 8.2, *p* = 0.046; populations within hosts: *λ* = 0.26, approx. *F*_*4*,*75*_ = 5.9, *p* = 0.0004). This multivariate difference appeared to be mainly due to variation in hydrocarbon component 13Me27 (82% variation between hosts, as opposed to 14% variation between populations within hosts, p = 0.048). None of the remaining hydrocarbon components displayed significantly more variation between populations on different hostplants as compared to populations from the same host-plant.

## Discussion and conclusion

We have used a combination of data from female cuticular hydrocarbon profiles, interspecific mating trials, and phylogenetics to evaluate the potential role of cuticular hydrocarbons in mating and diversification of *Timema* walking sticks. Our primary findings are that (1) hydrocarbon profiles differ substantially between species, and between *T*. *cristinae* populations adapted to different host plants, such that species-specific and population-specific sets of hydrocarbons may serve as signals for mate choice between species and populations; (2) in no-choice trials, mating was significantly more likely between pairs of species with similar hydrocarbon profiles than between species with divergent profiles; and (3) the macroevolution of major components of hydrocarbon profiles (DF1) fits best with a model of punctuational phenotypic change, as expected under change driven by selection processes. Taken together, these convergent lines of evidence strongly suggest a role for cuticular hydrocarbon variation in mate choice and speciation in the genus *Timema*, although elucidating the exact nature of this role will require additional studies. Notably, the experimental manipulation of hydrocarbon profiles are required to demonstrate that these profiles are indeed used as mating cues in *Timema*, and will allow direct assessment of the importance of hydrocarbons for mating preferences at the individual level and at different degrees of reproductive isolation.

Among other insects, cuticular hydrocarbon variation has been implicated in a range of developmental, ecological and behavioral contexts, including ecological adaptation to the local abiotic or biotic environment [40], effects from temperature and nutrition during juvenile stages [41,42], interactions with host plants [43,44], and mate choice and sexual selection within species [45,46]. Evidence for cuticular hydrocarbons mediating the evolution of reproductive isolation has thus far centered mainly on associations between variation in hydrocarbon profiles and variation in propensities to mate [47]. For example, in *Chorthippus parallelus* grasshoppers, male hydrocarbon variation, but not other potential mate-choice signals, was significantly linked with the degree of assortative mating among individuals from 12 populations [48]. Similarly, in *Chrysochus* beetles [49], and some *Drosophila* vinegar flies (e. g., [50,51]), mating between individuals from closely-related species or strains is affected in part by variation in cuticular hydrocarbon profiles. In *Timema*, assuming individuals indeed detect aspects of hydrocarbon profiles, decreased propensity to accept mates with lower hydrocarbon profile similarity would limit the likelihood of potentially costly interspecific matings. Therefore, cuticular hydrocarbon profiles and preferences may act as effective isolation mechanisms among divergent and potentially post-zygotically incompatible populations and species. Alternatively, reproductive isolation, and cuticular hydrocarbon profiles, may evolve as byproducts of species divergence. The precise roles of hydrocarbon variation in processes underlying the evolution of reproductive isolation, and the timing of evolutionary changes in hydrocarbon profiles during the process of speciation, remain to be elucidated, and require integration of data on selective pressures, mechanisms, and underlying genetic changes from different temporal and spatial scales.

In this study, we have analyzed cuticular hydrocarbon variation across nine species, and multiple populations, at varying levels of phylogenetic and genetic divergence. Inference of the tempo and mode of hydrocarbon profile evolution provides evidence generally consistent with the hypothesis that change in this set of characters does not occur gradually during phyletic evolution, but relatively rapidly, in proportion to the number of speciation events. This pattern of speciational change has also been found for the macro evolution of *Timema* courtship behavior, but not for the macroevolution of male genitalic morphology, which has diversified in proportion to phyletic branch lengths, apparently under forces of sexual selection or sexual conflict [52]. Among other insect species, previous studies of macroevolutionary change in hydrocarbon profiles have reported rapid evolution in conjunction with species specificity and a notable degree of phylogenetic conservatism [50,53,54], patterns that are largely concordant with those described here for *Timema*, but have not been partitioned into speciational versus phyletic concentrations of change. If changes in cuticular hydrocarbon profile drive speciation, then directional selection due to mate choice with profile-based criteria should characterize processes of divergence or reinforcement, while stabilizing selection should predominate at other times. Alternatively, hydrocarbon profiles may diverge between populations primarily due to differences in ecological variables such as temperature, humidity, habitat preference, or host-plant use, promoting the evolution of pre- and post-zygotic isolation under circumstances where such profiles also function in intraspecific or interspecific mate choices [29,40].

In *Timema*, natural selection for cryptic coloration patterns that match those of their host plants represents a major force in phenotypic divergence among populations and species [34,55-57]. Our intraspecific data showing hydrocarbon profile differences between *T*. *cristinae* from their two host plants, *Adenostoma* and *Ceanothus*, which are distributed in large- and small-scale mosaic patterns across their chaparral habitat, indicate that hydrocarbon-based chemical signals could be used as indicators of hostplant and other environmental adaptations [58] of a prospective mating partner. In our interspecific analyses of hydrocarbon profiles, we also found significant differences between the two populations sampled for *T*. *poppensis* and *T*. *chumash* (Figure 1). In these cases, the study of additional populations from each host plant may indicate whether these profile divergences are also correlated to specific hostplant adaptations as in *T*. *cristinae*.

The degree to which cuticular hydrocarbon profiles are genetically-based, compared to being acquired from the host plant or other features of the local environment, remains to be investigated in *Timema*; both heritable and environmental effects have been well established among other insects (e. g., [29,40,59]). Recent studies have identified sets of genes, mainly desaturases, that underlie variation in hydrocarbon profiles among *Drosophila*. Findings of rapid evolution and positive selection of such genes [60,61] suggest that hydrocarbon evolution may exert strong, direct effects on phenotypic changes that mediate speciation. In *Timema*, the rapid changes in CHC profiles associated with speciation events and their correlation with reproductive isolation, provide further support for the idea that such traits can play an integral role in population divergence and speciation.

## Methods

### Insect collection and mating trials

We collected data on intra- and interspecific mating propensity, hydrocarbon profile variation and genetic divergences for nine of the 16 described sexual *Timema* species (*T*. *bartmani*, *T*. *boharti*, *T*. *californicum*, *T*. *chumash*, *T*. *cristinae*, *T*. *knulli*, *T*. *petita*, *T*. *podura*, and *T*. *poppensis*). These species include all but one sexual species from three subclades in the *Timema* genus, mainly distributed in central California. The six remaining sexual species not included in this study, from southernmost California, Arizona, and Mexico, are basal to these subdivisions [38,62]. Such a near-complete coverage of species in a clade is necessary for valid tests of speciational change hypotheses, as missing species introduce errors into estimations of the number of speciation events separating a given species pair [19]. Another factor affecting the inference of speciation events are species extinctions, which are assumed to be either negligible [63] or to have occurred at random on the phylogeny [19,64].

Insects were collected using sweep nets between March and May of 2008 to 2011. Only individuals collected as juvenileswere used for analyses, and males and females were housed separately on their original host plant and raised to adults. This protocol ensured that all individuals used in experiments were virgins and of similar age. Different *Timema* species occur over latitudinally and altitudinally spread locations, such that the average developmental stage (number of molts to maturity) of individuals at a given date may vary greatly among populations. For the mating trials we needed individuals of each species that reached maturity at approximately the same time. For population combinations where developmental stages were very different, we therefore maintained juvenile males and females of the more advanced population in the refrigerator at 7°C for up to one week to slow down their development. Even though this treatment is unlikely to influence our results given only early juvenile stages were concerned, we nevertheless used these individuals only for mating trials, not for other experiments.

No-choice mating trials were conducted by introducing one male and one female into a 6-cm Petri dish. As in previous studies (e.g., [30,33]), we recorded after 1 h whether the pair was copulating. Mating proportions for each male-species by female-species combination were then estimated as the proportion of copulating pairs out of the total number of trials conducted for that combination. Across the nine species (81 pairwise combinations) we conducted >1400 no-choice trials (Table 2).

### Characterization of cuticular hydrocarbons

For each location, cuticular hydrocarbons of three to six adult virgin females (not used in the mating trials) were analyzed. Live individuals were anaesthetized by freezing for 1h, and submerged singly in 1 ml of HPLC-grade hexane for 5 min to extract the cuticular hydrocarbons from their body surface. The supernatant of each sample was then withdrawn, concentrated to circa 100 μl, and (*E*9)-octadecen-1-yl acetate (2 μl of a 100 ng/μl solution) was added as an internal standard (IS) for quantitative analyzes. The total amount of each target cuticular hydrocarbon (see below) was determined by multiplying the area count of the respective chromatographic peak (see below) with the 200 ng of the IS and by dividing the product by the area count of the IS.

Samples were analyzed with a Varian 3800 gas chromatograph (GC) coupled to a Varian Saturn Ion Trap mass spectrometer (MS) equipped with a DB-5 MS column (50 m ×0.25 mm i.d. for analyzes in 2009 and 2010; 30 m ×0.25 mm i.d. for analyzes in in 2011), using the following temperature program: 50°C for 2 min, then 20°C per min to 280°C (in 2009 and 2010) or to 240°C (in 2011). The final temperature of 280°C or 240°C was held for 46 min or 28 min, respectively. The injector temperature was 300°C. The mass spectrometer was set to scan for fragment ions between *m*/*z* 41 to *m*/*z* 500.

In 2009, analyses of body surface extracts of *T*. *cristinae*, *T*. *knulli* and *T*. *poppensis* revealed quantitative and qualitative differences in cuticular hydrocarbons between species, especially components eluting between heptacosane and octacosane. These components were selected as potential indicators of species-specific profiles. The components were hypothesized to be 3-, 5-, 7-, 9-, 11- and 13-methylheptacosane (henceforth 3Me27, 5Me27, 7Me27, 9Me27, 11Me27 and 13Me27, respectively) based on diagnostic fragment ions of their mass spectra [65]. The presence of these fragment ions was confirmed by re-analyzes of the samples on a Hewlett Packard GCD Quadrupole mass spectrometer fitted with a DB-5 column (30 m × 0.25 mm i.d.), scanning for fragment ions between *m*/*z* 41 and *m*/*z* 425. To confirm the structural assignment for each of these components, previously known 3Me27 [66], 5Me27 [67,68], and 7Me27, 9Me27, 11Me27 and 13Me27 [68,69] were synthesized by coupling methylketones with phosphorus ylides, and by hydrogenating the resulting olefins. Identical mass spectra and retention times of each of the insect-produced and corresponding synthetic components confirmed all structural assignments. Because in a large number of samples it was not possible to completely separate 9Me27 and 11Me27, we pooled them for further analyses.

We analyzed proportional rather than absolute abundances of components, to remove species differences stemming from body size variation (up to threefold among *Timema* females) and to reduce experimental error [14,70]. Proportional cuticular hydrocarbon components were calculated by dividing the amount of each component in a given sample by the sum of all components in that sample. These hydrocarbon proportions were then transformed using log-contrasts [14,36] to remove the non-independence among analyzed variables. Log contrasts were calculated by dividing the value for each hydrocarbon by the component 13Me27, and then taking the log of these new variables, resulting in four log-contrast transformed values [log-contrasts for 3Me27, 5Me27, 7Me27 and 9/11Me27, referred to as LC3 (Log Contrast 3), LC5, LC7 and LC9-11, respectively] for every individual. Results using other components as the divisor were qualitatively similar.

To test for species and population differences in hydrocarbon profiles we performed a multivariate analysis of variance (MANOVA; [71]), with populations nested within species. To test for significance of the main factor (species) against the nested term, we used the nested.npmanova command in the BiodiversityR package [72] which evaluates the statistical significance of the *F*-ratios by permutation. To test for the effect of species, populations were randomized between species. To test for the effect of populations, individuals were randomized among populations. Given these permutation schemes, the indicated p-values correspond to the proportion of randomized datasets producing a larger or equal F-ratio than the original dataset. We calculated p-values for 1000 randomizations. Populations within each individual species were compared using parametric MANOVAS (based on Wilk’s test statistic).

The expression of hydrocarbon profiles as well as other signal traits, are known to be affected by environmental influences as well individual condition (e.g., [43,73,74]). While our sample of two populations per species, from different host plants in most cases, allows us to assess species differences while including intra-specific variation, the sampling scheme is not designed to investigate the influence of environmental effects. Thus, future work will be required to distinguish the relative contribution of plastic effects, especially those linked to host plant rearing environment, versus genetic influences on cuticular hydrocarbon profiles (see for example [43]).

To test whether interspecific matings were more likely between species with similar than divergent hydrocarbon profiles, we estimated profile divergences between species using the multivariate Euclidian distance with the species median values for each individual hydrocarbon component as a reference. In other words, each hydrocarbon component defines an axis in a multi-dimensional space, in which each species is represented by a cloud of points – the Euclidian distance between two species is the distance between the two cloud centers (given by the median value on each axis) in the multi-dimensional space. We used (partial) Mantel tests implemented in the R package vegan1.17-4 [75] to test whether this distance is correlated with interspecific mating propensity (proportion of between species trials resulting in successful copulation) and genetic distance.

### Phylogenetic analyses

We next wanted to develop insights into the processes that shape hydrocarbon profile evolution among sexual *Timema* species. To this end, we evaluated the fit of different diffusion-based maximum-likelihood models, each representing a specific evolutionary scenario. In particular, we wanted to infer whether hydrocarbon profiles tend to diverge continuously between species, or whether profile changes occur in proportion to speciation events. These evolutionary scenarios can be evaluated by applying different evolutionary models of character change in a phylogenetic framework [19]. We used the program CoMET [76] within the Mesquite system [77] to infer which of nine different character change models best describes the diversification of hydrocarbon profiles in *Timema*. Specifically, the nine models implemented in CoMET represent all possible combinations of three different phylogenetic models with three different tempo-of-change models, in a 3 × 3 matrix [76] (Additional file 1). The phylogenetic models describe the pattern of character change as pure-phylogenetic, non-phylogenetic or punctuational. Under the pure-phylogenetic model, character change occurs along all branches in the phylogeny so that the level of character divergence is correlated with the level of phylogenetic divergence. The non-phylogenetic model assumes a star phylogeny, which means that character divergence occurs independently of phylogenetic divergence. The punctuational model assumes that at each bifurcation in the tree, one daughter branch retains the ancestral character value, and the other daughter branch changes. The tempo-of-change models evaluate three different rates (distance, equal and free) at which the character can change on each branch. In the distance model, change is proportional to genetic distance (i.e., branch length). The equal model assumes that all branches in the phylogeny have the same lengths, and phenotypic change is therefore independent of time since divergence. In the free model, the length of each branch is proportional to the amount of character change occurred, rather than to genetic divergence. Under this model, the characters change at different rates in different lineages, and character change is thus not proportional to time since divergence. Overall, CoMET thus estimates the fit of nine distinct models of character change to the *Timema* phylogeny (Additional file 1), whereby Akaike Information Criterion (AIC) values are calculated to determine the model of evolutionary change that best characterizes the tempo and mode of diversification of hydrocarbon profiles in *Timema*. Note that while second order information criterion (AICc) values are usually preferred over AIC values for model selection (because under AICc, the relative penalty for model complexity increases for small datasets; [78]), AICc cannot be applied for the free rates tempo-of-change models, given the number of parameters estimated in these models (see Additional file 1 for details). This issue has, however, no influence on our conclusions as even with the less stringent penalizing for model complexity, we find the best fit for the simple models (in which only one parameter is estimated; see results).

Several previous studies have focused on species relationships in *Timema*[38,39]. However, because the model evaluation with CoMET requires character values for each tip in the phylogeny, we built a new phylogeny using the nine species for which we determined hydrocarbon profiles and interspecific mating propensities. This phylogeny was based on concatenated mitochondrial *COI* and nuclear *Hsp70* sequences (total of 1879 bp) that we generated for a previous study (see Additional file 2 for GenBank accession numbers). Maximum likelihood phylogenetic analyzes (with heuristic tree searches) were carried out using PAUP*4.0b10 [79] on the freely available Bioportal (http://www.bioportal.uio.no). We used the optimal model of sequence evolution (GTR+I+G) identified with the Akaike information criterion (AIC) as implemented in jMODELTEST 0.1.1 [80]. Branch support was evaluated by a maximum-likelihood bootstrapping analysis using Seqboot (500 replicates), DNAml, and Consense within the Phylip 3.68 package [81]. For completeness, we also inferred Bayesian posterior probabilities with MrBayes3.1.2 [82], with Markov chains run for 10^6^ generations, 10^4^ generations burn-in values, and trees sampled every 100 generations.

To test whether hydrocarbon profile distances are also correlated with intraspecific mating propensities, we used four additional *T*. *cristinae* populations. *Timema cristinae* populations are morphologically adapted to different host plants (*Adenostoma* and *Ceanothus*; [83]), and there is extensive evidence for weak to moderate levels of sexual isolation between populations on different hosts [32-34]. However, sexual isolation is not based on morphology [84], and thus further work on the causes of sexual isolation is warranted. For logistic reasons, it was not possible to use the same methods as described for analyses of interspecific hydrocarbon profiles. Instead of using lab-raised virgin females, we directly collected 30 adult females from two *Ceanothus* populations and 41 from two *Adentostoma* populations. Although females thus represent a pool of different ages and mating status, this protocol is more likely to introduce error than systematic bias in terms of population divergence in hydrocarbon profiles. Cuticular hydrocarbons of each individual were directly extracted in the field and stored on ice until they were analyzed as described above. Because it was not possible to objectively separate 11Me27 and 13Me27 in these field samples, the two components were pooled; all remaining analyzes were conducted as described for interspecific comparisons.

## Availability of supporting data

Data archived in the DRYAD repository under doi:10.5061/dryad.98f8c.

## Competing interests

The authors declare that they have no competing interests.

## Authors’ contributions

TS and BJC planned the study, RG and GG performed hydrocarbon experiments, DA and TS conducted interspecific mating tests, TS analyzed the data, TS and BJC wrote the manuscript with input from all authors. All authors read and approved the final manuscript.

## Supplementary Material

Additional file 1 **Details on the nine models of character evolution evaluated on the *Timema *phylogeny for the different hydrocarbon components and global profiles.** Click here for file

Additional file 2 **Table with GenBank accession numbers for sequences used to build the *Timema *phylogeny.** Click here for file
