# Supplementary material for: Hydrocarbon divergence and reproductive isolation in Timema stick insects
Source: BMC Evol Biol. 2013 Jul 16;13:151. doi: 10.1186/1471-2148-13-151 (PMC3728149; doi:10.1186/1471-2148-13-151)
Supplement: Additional file 1 — Details on the nine models of character evolution evaluated on the Timema phylogeny for the different hydrocarbon components and global profiles. [file 1471-2148-13-151-S1.docx]

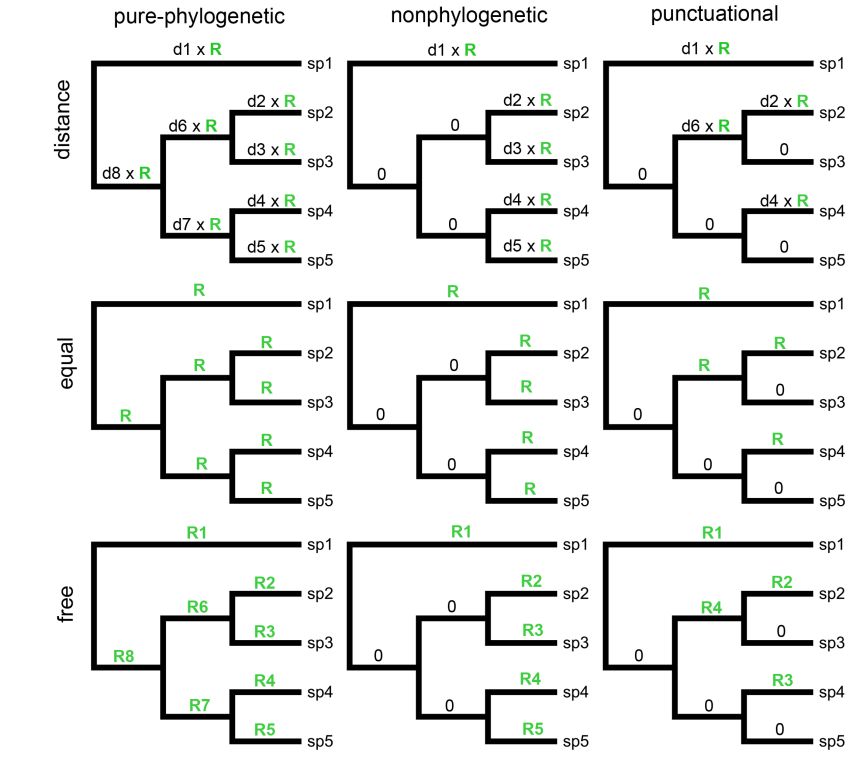


**Additional File 1**, Figure adapted from Oakley et al. (2005). The nine models of character evolution evaluated by CoMET are combinations of three different phylogenetic models (pure phylogenetic, nonphylogenetic, punctuational) with three different tempo-of-change models (distance, equal, free). The pure phylogenetic models assume that character change occurs on every branch of the phylogeny, the nonphylogenetic models assume that there is no phylogenetic component to trait evolution (i.e., a star-shaped phylogeny), and the punctuated class assumes character change occurs on only one of every pair of descendent branches. Labels on branches symbolise the amount of change occurring on each branch, whereby change is proportional to genetic distances (d1-d8 given by the species phylogeny) in the distance models (top row). Parameters estimated from the data are indicated in green (R, R1-R8); only a single parameter (R) is estimated for each of the distance and equal models. The number of estimated parameters for the free models depends on the number of species in the phylogeny (N); 2N-2 parameters are estimated for the pure-phylogenetic/free model, N for the nonphylogenetic/free model and N-1 for the punctuated/free model.
