# Supplementary material for: Hydrocarbon divergence and reproductive isolation in Timema stick insects
Source: BMC Evol Biol. 2013 Jul 16;13:151. doi: 10.1186/1471-2148-13-151 (PMC3728149; doi:10.1186/1471-2148-13-151)
Supplement: Additional file 2 — Table with GenBank accession numbers for sequences used to build the Timema phylogeny. [file 1471-2148-13-151-S2.docx]

**Additional File 2**. Genbank accession numbers for sequences used to build the *Timema* sexual species phylogeny.

| Species | *COI* Sequences | *HSP70* Sequences |
| --- | --- | --- |
| *T. bartmani* | HQ184475-487 | HQ198154-159 |
| *T. boharti* | HQ184488-491 | HQ198160-163 |
| *T. californicum* | JX237536-546 | HQ198164-171 |
| *T. chumash* | HQ184519-529 | HQ198172-175 |
| *T. cristinae* | HQ184530-542 | HQ198176-187 |
| *T. knulli* | JX237530-531 | JX237557-559 |
| *T. petita* | JX237532-535 | JX237554-556 |
| *T. podura* | HQ184597-610 | HQ198216-223 |
| *T. poppensis* | HQ184611-657 | HQ198224-235 |
